# Relative resistance of HIV-1 founder viruses to control by interferon-alpha

**DOI:** 10.1186/1742-4690-10-146

**Published:** 2013-12-03

**Authors:** Angharad E Fenton-May, Oliver Dibben, Tanja Emmerich, Haitao Ding, Katja Pfafferott, Marlen M Aasa-Chapman, Pierre Pellegrino, Ian Williams, Myron S Cohen, Feng Gao, George M Shaw, Beatrice H Hahn, Christina Ochsenbauer, John C Kappes, Persephone Borrow

**Affiliations:** 1 Nuffield Department of Medicine, University of Oxford, NDM Research Building, Old Road Campus, Headington, Oxford OX3 7FZ, UK; 2 Department of Medicine, University of Alabama at Birmingham, Birmingham, AL, USA; 3 Division of Infection and Immunity, Faculty of Medicine and Centre for Sexual Health & HIV Research, London, UK; 4 Centre for Sexual Health & HIV Research, Mortimer Market Centre, University College London, London, UK; 5 Department of Medicine, University of North Carolina at Chapel Hill, Chapel Hill, NC, USA; 6 Duke Human Vaccine Institute and Department of Medicine, Duke University Medical Centre, Durham, NC, USA; 7 Departments of Medicine and Microbiology, Perelman School of Medicine, University of Pennsylvania, Philadelphia, PA, USA; 8 Birmingham Veterans Affairs Medical Center, Research Service, Birmingham, AL, USA

**Keywords:** Human immunodeficiency virus type 1, Type 1 interferon, Viral inhibition, Founder virus, Acute infection

## Abstract

**Background:**

Following mucosal human immunodeficiency virus type 1 (HIV-1) transmission, type 1 interferons (IFNs) are rapidly induced at sites of initial virus replication in the mucosa and draining lymph nodes. However, the role played by IFN-stimulated antiviral activity in restricting HIV-1 replication during the initial stages of infection is not clear. We hypothesized that if type 1 IFNs exert selective pressure on HIV-1 replication in the earliest stages of infection, the founder viruses that succeed in establishing systemic infection would be more IFN-resistant than viruses replicating during chronic infection, when type 1 IFNs are produced at much lower levels. To address this hypothesis, the relative resistance of virus isolates derived from HIV-1-infected individuals during acute and chronic infection to control by type 1 IFNs was analysed.

**Results:**

The replication of plasma virus isolates generated from subjects acutely infected with HIV-1 and molecularly cloned founder HIV-1 strains could be reduced but not fully suppressed by type 1 IFNs *in vitro*. The mean IC_50_ value for IFNα2 (22 U/ml) was lower than that for IFNβ (346 U/ml), although at maximally-inhibitory concentrations both IFN subtypes inhibited virus replication to similar extents. Individual virus isolates exhibited differential susceptibility to inhibition by IFNα2 and IFNβ, likely reflecting variation in resistance to differentially up-regulated IFN-stimulated genes. Virus isolates from subjects acutely infected with HIV-1 were significantly more resistant to *in vitro* control by IFNα than virus isolates generated from the same individuals during chronic, asymptomatic infection. Viral IFN resistance declined rapidly after the acute phase of infection: in five subjects, viruses derived from six-month consensus molecular clones were significantly more sensitive to the antiviral effects of IFNs than the corresponding founder viruses.

**Conclusions:**

The establishment of systemic HIV-1 infection by relatively IFNα-resistant founder viruses lends strong support to the hypothesis that IFNα plays an important role in the control of HIV-1 replication during the earliest stages of infection, prior to systemic viral spread. These findings suggest that it may be possible to harness the antiviral activity of type 1 IFNs in prophylactic and potentially also therapeutic strategies to combat HIV-1 infection.

## Background

Events in the acute phase of human immunodeficiency virus type 1 (HIV-1) infection play a critical role in determining the subsequent disease course, and are therefore important to characterise in order to facilitate the rational development of strategies for HIV prophylaxis or therapy. Initial HIV-immune system interactions at mucosal exposure sites dictate whether the transmitted virus is eliminated or undergoes sufficient local expansion to enable dissemination to local lymphoid tissues [1]. The observations that a high number of exposures are typically required for heterosexual HIV transmission and that disseminated infection is frequently initiated by a single founder virus [2,3] suggest that rapidly-activated local responses may extinguish the initial foci of replication established by the majority of virions before widespread dissemination occurs. If infection does spread, systemic immune responses impact the magnitude of the acute viremic burst and associated CD4 T cell destruction, and influence the subsequent efficiency of control of viremia [4]. Adaptive responses start to be induced as widespread HIV replication occurs and are known to contribute to containment of the acute viremic burst and influence the persisting viral load established thereafter [4]. By contrast innate responses, which are activated much more rapidly, have the capacity to impact viral replication from the earliest stages of infection and may have an even greater effect on the outcome of infection [5]. However the contribution of innate responses to early control of HIV replication is much less well understood.

Type 1 interferons (IFNs) are a family of innate cytokines that includes IFNβ and 13 subtypes of IFNα in humans. They are constitutively produced at very low levels, but can be rapidly up-regulated in response to pathogen exposure or infection and play important effector and immunoregulatory roles in the early host immune response. Type 1 IFNs mediate their activity by binding to the IFNα/β receptor, which is expressed on all nucleated cells. This, in turn, induces the up-regulation of interferon-stimulated genes (ISGs) [6]. There are hundreds of IFN-responsive genes, the functions of many of which remain to be elucidated, whilst others are known to exert direct antiviral activity or regulate the activation state, functions, proliferation or survival of host cells [7]. Type 1 IFNs are therefore highly pleiotropic innate cytokines that can contribute to viral containment by both direct and indirect mechanisms [8]. Their importance in early viral control is indicated by the plethora of strategies that viruses have evolved to impair the production or activity of type 1 IFNs [9-11] and has been demonstrated experimentally using IFN-blocking antibodies and IFNα/β receptor-deficient mice [12,13]. However, chronic production of type 1 IFNs during persistent viral infections can have detrimental effects, driving hyperimmune activation and impairing control of ongoing viral replication [14,15].

Up-regulation of type 1 IFN production is one of the earliest innate responses observed in HIV-1 infection. The innate responses activated at mucosal sites of HIV exposure are difficult to analyse in humans, but studies performed in macaques infected intravaginally with simian immunodeficiency virus (SIV) suggest that plasmacytoid dendritic cells (pDCs) can be recruited to mucosal sites and become activated to produce high levels of IFNα and β within 24 hours of virus exposure [16]. Subsequent SIV spread to lymphoid tissues is likewise accompanied by up-regulation of IFNα and β production at these sites [17]. In humans, systemic virus dissemination is also associated with rapid type 1 IFN production: as plasma virus titres increase during acute HIV infection there is a transient elevation in circulating levels of IFNα that peaks prior to the peak in viremia [18]. The concomitant drop in circulating pDC numbers [19], reflecting pDC activation and recruitment into lymphoid tissues [20], suggests that pDCs probably constitute the major cellular source of type 1 IFN in tissues and plasma.

Type 1 IFNs are known to mediate control of HIV replication in both CD4 T cells and macrophages *in vitro*[21,22]. They act to inhibit HIV replication at multiple stages in the viral lifecycle [23,24], suggesting that an array of ISGs acting via different mechanisms is involved in mediating antiviral activity against HIV. Consistent with this, multiple ISGs that restrict HIV-1 replication by diverse mechanisms have been identified, including apolipoprotein B mRNA-editing, enzyme-catalytic, polypeptide-like (APOBEC)3G/3F [25-27], tripartite motif-containing protein (TRIM) 5α [28,29], tetherin [30,31], schlafen 11 [32] and more broadly-acting antiviral ISGs such as protein kinase R (PKR) [33,34], interferon-induced transmembrane (IFITM) proteins [35,36], and ISG15 [37].

The potential for type 1 IFNs to restrict HIV replication is indicated by the fact that HIV has evolved strategies for limiting type 1 IFN induction during infection and for counteracting the activities of ISGs. Most cells express cytosolic nucleic acid receptors that detect viral DNA or RNA that, if the cell becomes infected, trigger interferon-responsive factor (IRF)3 activation, which up-regulates type 1 IFN production. Notably, HIV infection of T cells and macrophages does not elicit type 1 IFN production by these cells [38,39]. Several complementary mechanisms may account for this: the host exonuclease three prime repair exonuclease 1 (TREX1) degrades HIV DNA, avoiding HIV detection by cytosolic DNA sensors [40]; the HIV protease targets the cytosolic RNA receptor retinoic acid-inducible gene 1 (RIG-1), which is capable of sensing HIV-1 RNA, to lysosomes where it is degraded [41]; and Vpu impairs triggering of NFκB activation via cytosolic RNA and DNA receptors [42-44]. Although these mechanisms limit type 1 IFN production by infected cells, high levels of type 1 IFN production still occur after HIV-1 infection *in vivo*. This is because specialised cells such as pDCs that can produce type 1 IFNs following exposure to virus without themselves becoming infected are triggered to produce high levels of type 1 IFNs by HIV or HIV-infected cells [45,46]: pDCs likely constitute a major source of type 1 IFN production during acute HIV-1 infection. HIV-1 has also evolved multiple mechanisms for evading control by antiviral ISGs, for example Vif antagonises APOBEC3G/F activity [25,47], the HIV-1 capsid gives the virus a low sensitivity to human TRIM5α [28,29], Vpu counteracts the effects of tetherin [30,31,48-50] and binding of Tat to the HIV-1 transactivation response (TAR) element inhibits the intrinsic ability of TAR to stimulate activation of PKR and 2’ ,5’-oligoadenylate synthase (2’ ,5’-OAS) [33,51,52].

Although these IFN evasion strategies implicate type 1 IFN-mediated antiviral activity as a major selective force during HIV-1 evolution, the importance of type 1 IFN-mediated antiviral activity in *in vivo* control of the virus remains unclear. Administration of IFNα to individuals chronically infected with HIV has been associated with some improvement in viral control [53-58], although the effects were generally modest, and the roles of direct IFN-mediated antiviral activity versus IFN-induced enhancement of HIV control via stimulation of other immune responses have not been dissected. Most importantly, the contribution of type 1 IFNs to HIV-1 control in the earliest stages of infection has not been addressed. Mucosal administration of toll-like receptor (TLR) agonists prior to intravaginal or intrarectal SIV challenge in macaques did not result in a significant decrease in (and in one study in fact enhanced) early virus replication [59,60]; however these studies are difficult to interpret as TLR ligands do not solely up-regulate type 1 IFNs, but also have other activities including induction of proinflammatory cytokines.

We previously demonstrated that HIV-1-specific CD8 T cell and neutralizing antibody responses can exert biologically significant pressure on *in vivo* viral replication by demonstrating rapid emergence of viruses with enhanced resistance to control by these adaptive responses in infected individuals [61,62]. Here, we used a similar approach to determine whether type 1 IFNs exert selective pressure on HIV-1 replication during the initial stages of infection. We hypothesised that if the direct antiviral activity of type 1 IFNs plays a significant role in control of HIV-1 replication during the establishment of infection, viruses with a relatively high sensitivity to IFN-mediated antiviral activity would have a reduced capacity to establish a productive infection. In contrast, the founder viruses that succeeded in undergoing amplification in the presence of high levels of type 1 IFN would tend to be relatively resistant to IFN-mediated antiviral activity; and would be expected to be more IFN-resistant than viruses present during chronic infection, when type 1 IFNs are produced at much lower levels and ongoing virus replication is not subject to similarly-strong IFN-mediated pressure. To test this hypothesis, we established an *in vitro* assay for assessing the sensitivity of HIV-1 isolates to control by type 1 IFN-mediated antiviral activity, and used this to compare the relative IFN-sensitivity of viruses isolated from HIV-1-infected individuals at different stages of infection. Virus isolates from acutely-infected patients were found to be significantly more resistant to *in vitro* control by IFNα than virus isolates generated from the same patients during chronic, asymptomatic infection, supporting an important role for type 1 IFN-mediated antiviral activity in control of HIV-1 replication during the establishment of infection.

## Results and discussion

### Establishment of an experimental system for evaluation of HIV-1 sensitivity to the antiviral activity of type 1 IFNs

**Figure 1:**
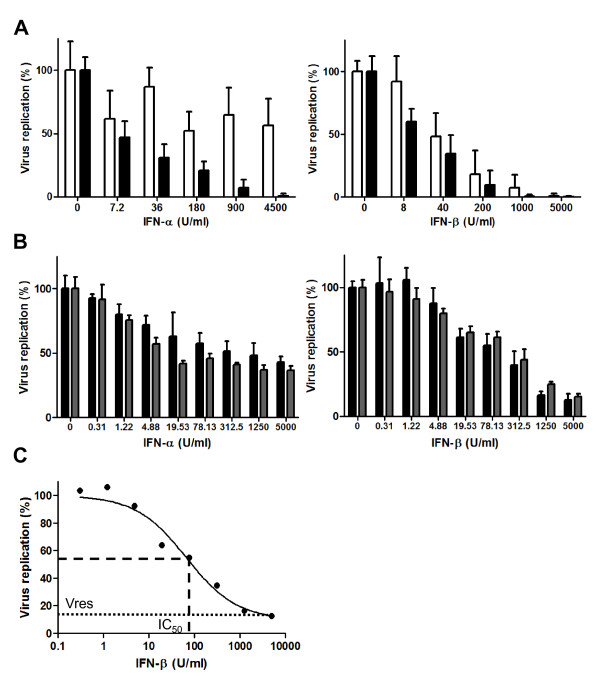
***In vitro *analysis of the inhibition of HIV-1 replication by IFNα and IFNβ. (A)** Comparison of the inhibition of HIV (W6BC) replication when cells were treated with IFNα or IFNβ for 4 hours prior to infection (open bars) or were treated with IFNα or IFNβ both prior to infection and throughout the subsequent viral replication period (filled bars). Viral replication was assessed by measurement of supernatant p24 levels on day 7 post-infection. The results shown are the mean p24 values from 5 replicate wells treated with the indicated concentrations of IFN, expressed as a % of p24 values from cells that were not IFN-treated. Error bars represent 1 standard deviation above the mean. **(B)** Example of data from assays performed to assess the inhibition of a representative primary HIV-1 isolate (generated from plasma cryopreserved during acute infection from subject MM38) by IFNα and IFNβ. Inhibition of virus replication by each IFN subtype was assessed in two independent assays using mixed PBMCs derived from different groups of donors (black and grey bars). The results shown are the mean p24 values from 4 replicate wells treated with the indicated concentrations of IFN, expressed as a % of p24 values from cells that were not IFN-treated. Error bars represent 1 standard deviation above the mean. **(C)** Calculation of Vres and IC_50_ values from a representative IFN inhibition assay (the first IFNβ assay performed on the acute time-point virus isolate from subject MM38 in B). The level of viral replication in the presence of IFNβ (mean supernatant p24 concentration, expressed as a percentage of the mean p24 concentration in the absence of IFN) is plotted against the IFNβ concentration and a curve fitted to the data by non-linear regression using a least squares method. The Vres value (level of virus replication observed in the presence of maximally-suppressive IFN concentrations) is indicated by the dotted line. The IC_50_ value (IFN concentration required to produce half-maximal inhibition of viral replication i.e. midway between 100% replication and Vres) is read off from the inhibition curve as indicated by the dashed line.

To quantitate the relative sensitivity of different HIV-1 isolates to type 1 IFN-mediated antiviral activity, we established an *in vitro* assay to evaluate the extent to which virus replication in activated CD4+ cells derived from the peripheral blood of HIV-seronegative donors (chosen to mimic the cellular substrate in which the majority of productive virus replication occurs *in vivo*) was reduced by exposure of the cells to a range of concentrations of exogenously-added recombinant IFNα2 or IFNβ. Activated, CD8-depleted peripheral blood mononuclear cells (PBMCs) (a mixture of cells from 3 different donors, to reduce inter-assay variability) were pre-treated with IFN for 4 hours (to allow up-regulation of ISG expression), infected with HIV-1 at a low multiplicity of infection (moi) (0.001 50% tissue culture infectious doses (TCID)_50_/cell), then viral replication was assessed 7 days later by measurement of supernatant p24 levels. The day 7 time-point was chosen because in pilot experiments addressing the kinetics of growth of a panel of primary HIV-1 isolates, supernatant p24 levels reached maximal levels between 7 and 10 days post-infection. Addition of blocking antibodies to IFNα and β to the culture medium of non-IFN-treated cells did not alter viral replication, indicating that HIV-1 was not eliciting significant levels of type 1 IFN production from infected cells (data not shown). However, exogenously-added IFNα and IFNβ inhibited viral replication in a dose-dependent fashion (Figure 1A and B). Replicate assays performed with the same virus using mixed cells derived from different groups of donors gave very similar results (Figure 1B).

Comparison of the inhibition of HIV replication observed when cells were treated with IFN for 4 hours prior to infection only, to that observed when cells were pre-treated with IFN and IFN was also included in the culture medium throughout the 7-day viral replication period, showed that IFNα needed to be present throughout the course of infection to mediate its full inhibitory effects. By contrast, pre-treatment of cells with IFNβ was sufficient to induce near-maximal inhibition of virus replication (Figure 1A). IFNβ is known to bind with higher affinity to both the IFNAR2 and IFNAR1 chains of the IFNα/β receptor than IFNα subtypes including IFNα2 [63,64], which likely explains why IFNα needed to be present throughout the assay. In subsequent experiments, IFNα and IFNβ were thus included throughout the entire course of the assay to enable viral sensitivity to the full antiviral activity of each cytokine to be evaluated.

Use of IFNα and β at a range of concentrations up to 5000 U/ml was found to be sufficient to achieve maximal suppression (indicated by a plateau in inhibitory activity) of most viruses, although the extent to which HIV-1 replication was suppressed at maximally-inhibitory IFN concentrations differed for different strains, as illustrated by the examples in Figures 1A and B. The level of viral replication at maximal IFNα and β concentrations (expressed as a percentage of viral replication in the absence of IFN) was termed Vres (Viral resistance to inhibition by maximal IFN concentrations). Higher Vres values reflect a higher level of resistance to IFN-mediated antiviral activity. The concentration of IFNα and β required to achieve 50% of the inhibition of virus replication observed at maximal IFN concentrations (IC_50_) could also be calculated for each virus (Figure 1C).

### Analysis of the sensitivity of virus isolates from patients acutely-infected with HIV to in vitro control by IFNα and IFNβ

Virus isolates were generated from 11 patients presenting with symptomatic primary HIV-1 infection, all of whom were infected with clade B viruses (patients MM23, MM24, MM25, MM26, MM27, MM28, MM33, MM34, MM38, MM39 and CH077, Table 1), by co-culture of plasma cryopreserved during acute infection with activated CD4+ cells derived from the peripheral blood of HIV-seronegative donors. The primary virus isolates were expanded by further growth in activated CD4+ T cells, and the infectious titres of the resulting stocks were determined by TCID_50_ assay. Viruses were subjected to a minimum number of *in vitro* passages to ensure that the isolates generated were as representative as possible of the *in vivo* plasma virus.

**Table 1:** Patients from whom virus isolates and/or infectious molecular clones were generated

**Patient ID**	**Transmission risk group**^**1**^	**Clade**^**2**^	**Setpoint VL (copies/ml)**^**3**^	**Virus isolation time-points (DFOSx)**^**4**^	**IMCs generated**^**6**^
CH040	MSM	B	13,224	None	Founder^7^
CH058	MSM	B	260	28^5^	Founder, 6-month^7^
CH077	MSM	B	3,631	21^5^, 592^5^	Founder, 6-month^7^
CH162	HSX	C	114,815	None	Founder^8^
CH164	HSX	C	575,440	None	Founder^8^
CH185	HSX	C	40,738	None	Founder^8^
CH236	HSX	C	134,896	None	Founder, 6-month^9^
CH264	HSX	C	74,131	None	Founder^9^
CH470	MSM	B	23,442	33^5^	Founder, 6-month^10^
CH850	HSX	C	15,488	None	Founder, 6-month^9^
MM23	MSM	B	82,958	14, 204, 631, 1535	None
MM24	MSM	B	128,021	16, 1322	None
MM25	MSM	B	72,600	10	None
MM26	MSM	B	34,493	69	None
MM27	MSM	B	48,360	28, 1516	None
MM28	MSM	B	12,322	6, 1995	None
MM33	MSM	B	73,958	12, 1912	None
MM34	MSM	B	8,522	25, 2227	None
MM38	MSM	B	ND	29	None
MM39	MSM	B	8,546	11, 1206	None
SUMA	MSM	B	17,245	None	Founder^7^

^1^Transmission risk group: MSM = men who have sex with men; HSX = heterosexual.

^2^Clade of infecting virus: all clade B virus infected patients were recruited from clinical sites in the UK or USA, whilst clade C virus infected patients were recruited from clinical sites in Africa.

^3^Setpoint persisting viral load established, calculated as described by Fellay *et al.*[65].

^4^Time-points during infection when plasma from which virus isolates were subsequently derived was cryopreserved, expressed as days following onset of symptoms of the acute retroviral syndrome (DFOSx); or ^5^for those patients where the time of onset of symptoms was not known, expressed as days post Fiebig stage I/II of acute infection [66].

^6^HIV-1 IMCs generated: founder = IMC corresponding to the deduced sequence of the founder virus that established systemic infection; 6-month = IMC corresponding to the 6-month consensus virus sequence.

^7^SGA and viral sequence analysis reported in [67]; construction of the founder IMC described in [68]; construction of the 6-month IMC described in [69].

^8^SGA, viral sequence analysis and construction of the founder IMC described in [70].

^9^SGA and viral sequence analysis, unpublished data of G.M. Shaw and B.H. Hahn; founder and 6-month IMC construction, unpublished data of C. Ochsenbauer and J.C. Kappes.

^10^SGA, viral sequence analysis and construction of the founder IMC described in [70]; 6-month IMC construction, unpublished data of C. Ochsenbauer and J.C. Kappes.

**Figure 2:**
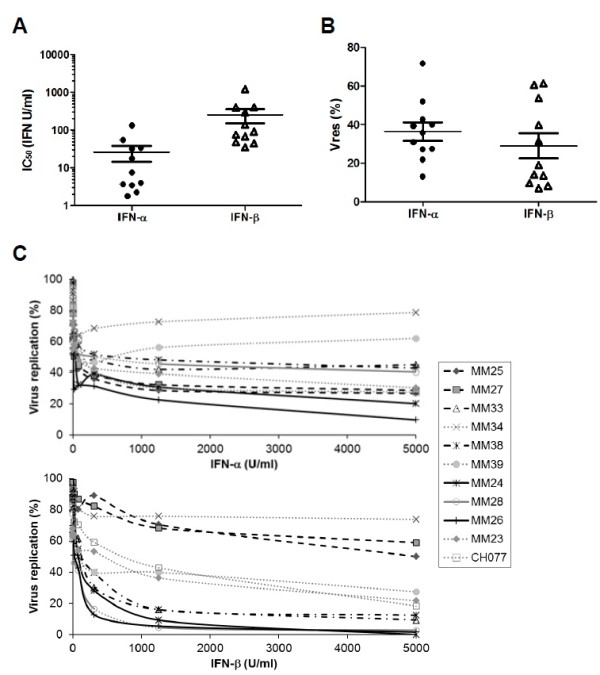
**Resistance of plasma virus isolates generated from subjects acutely infected with HIV-1 to *in vitro *control by IFN-α and IFN-β. (A)** IFNα (filled circles) and IFNβ (open triangles) IC_50_ values of virus isolates generated from different subjects during acute HIV infection; and **(B)** IFNα (filled circles) and IFNβ (open triangles) Vres values of these virus isolates. Each datapoint represents the mean (of results obtained in a minimum of 2 independent IFN inhibition assays) IC_50_ or Vres value calculated for a given virus isolate. The horizontal lines show the group mean IC_50_ or Vres values, plus and minus one standard error. **(C)** Inhibition of the replication of individual HIV-1 isolates at different IFN concentrations. The level of replication of each virus isolate in the presence of different concentrations of IFNα or IFNβ (mean supernatant p24 concentration, expressed as a percentage of the mean p24 concentration in the absence of IFN) was evaluated in a minimum of two independent assays, the mean of results from which is plotted against the IFN concentration, expressed on a linear scale to illustrate viral replication in the presence of high IFN concentrations. Clusters of virus isolates that exhibit shared patterns of relative resistance to control by IFNα and IFNβ are indicated by the bold solid, long-dashed and short-dashed lines.

The sensitivity of each virus isolate to *in vitro* control by IFNα and IFNβ was determined using the method described above: the IC_50_ and Vres values for the 11 viruses tested are shown in Figure 2A and B, respectively. The replication of all virus isolates could be inhibited to some extent by type 1 IFNs, but virus isolates from different patients exhibited differences in their IC_50_ and Vres values. Although there was overlap in the IC_50_ values for IFNα and IFNβ of this group of viruses, the mean IC_50_ value for IFNα (27 U/ml) was significantly lower than that for IFNβ (259 U/ml) (p = 0.0008, Mann–Whitney test), indicating that IFNα is capable of inhibiting HIV-1 replication at lower concentrations than IFNβ. However the range of Vres values observed for IFNα and IFNβ was very similar (13 - 72% for IFNα and 7 - 61% for IFNβ), showing that at high concentrations, IFNα and IFNβ are capable of suppressing virus replication to similar extents. During acute HIV-1 infection circulating levels of IFNα can reach several hundred U/ml [18,71], and it is likely that local concentrations of type 1 IFNs in tissues (where IFNβ exerts the majority of its activity) are higher still. This suggests that type 1 IFNs are up-regulated to levels sufficient to induce maximal suppression of HIV replication during acute infection. Nonetheless, it is likely that type 1 IFN concentrations rapidly become limiting during acute infection, as high-level type 1 IFN production occurs only transiently [18,71]. Thus, effective IFN-mediated control of HIV-1 replication may only be short-lived.

Interestingly, although viral Vres values for IFNα followed a Gaussian distribution, two clusters of viruses with relatively high and very low Vres values could be distinguished for IFNβ, indicating that there are a subset of viruses whose replication can be almost completely suppressed by IFNβ, although they are not controlled equally well by IFNα. Although IFNβ and IFNα subtypes bind to a common receptor, they signal differential patterns of ISG induction [7], which is thought to be due at least in part to differences in their receptor binding properties [63]. The group of highly IFNβ-sensitive viruses may be susceptible to control by one or more antiviral genes that is preferentially up-regulated by IFNβ compared to IFNα2. Likewise, as illustrated in Figure 2C, virus isolates sharing common patterns of resistance/sensitivity to IFNα versus IFNβ could be identified, likely reflecting a shared ability/lack of ability to resist control by differentially up-regulated antiviral ISGs.

### Investigation of the relationship between the IFN-sensitivity of acute virus isolates and the setpoint persisting viral load established in early infection

**Figure 3:**
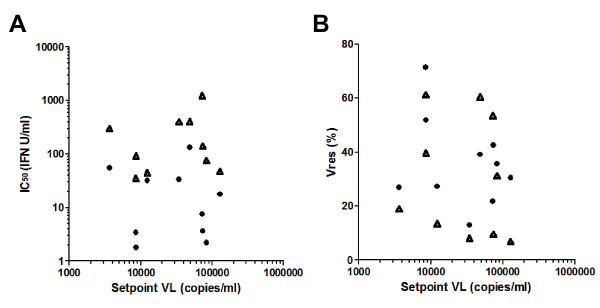
**Relationship between the IFN resistance of acute time-point virus isolates and the setpoint persisting viral load established in the subjects from which they were derived. (A)** IC_50_ values; and **(B)** Vres values of plasma virus isolates generated from different subjects during acute HIV infection are plotted against the setpoint persisting viral load subsequently established in the individual concerned in the absence of ART. Solid circles denote IC_50_ and Vres data for IFN-α and open triangles denote IC_50_ and Vres data for IFN-β. The data shown are the mean of results from a minimum of 2 independent IFNα or IFNβ assays performed with each virus using different donor PBMC mixes.

We reasoned that if type 1 IFN-mediated antiviral activity constitutes a major determinant of the magnitude of the acute burst of viral replication in primary HIV-1 infection and/or the efficiency of subsequent viral control, better HIV control and establishment of a lower viral setpoint may be observed in those patients in whom infection was established by more IFN-sensitive viruses. Ten of the 11 patients from whom virus isolates were generated during acute HIV-1 infection declined early antiretroviral therapy (ART), thus enabling calculation of their setpoint viral loads. As shown in Figure 3, there was no significant relationship between the Vres or IC_50_ values for either IFNα or IFNβ of the virus isolates generated from these subjects in acute infection and the setpoint viral loads they went on to establish. However, this analysis was limited by the relatively low number of subjects studied and the fact that the majority established moderate-high persisting viral loads, which is commonly observed in patients recruited on the basis of clinical presentation with symptomatic primary HIV-1 infection. Setpoint viremia is known to be determined by a combination of host and viral factors, including host genes in the chemokine receptor cluster (that affect viral entry) [72-76] and major histocompatibility region (that act at least in part by determining the efficiency of CD8+ T cell-mediated control of HIV replication) [65,73,77] and the replicative fitness of the infecting virus [78-80]. Given the multiplicity of factors involved in determining the viral setpoint, detection of any effects of viral IFN-resistance on setpoint viremia would likely require analysis of a much larger number of subjects. The results in Figure 3 therefore do not preclude a possible role for type 1 IFNs in restricting the magnitude of the acute viral burst and reducing the setpoint persisting viral load.

### Comparison of the in vitro IFN sensitivity of viruses isolated from patients acutely-infected with HIV and virus isolates generated from the same individuals during chronic infection

**Figure 4:**
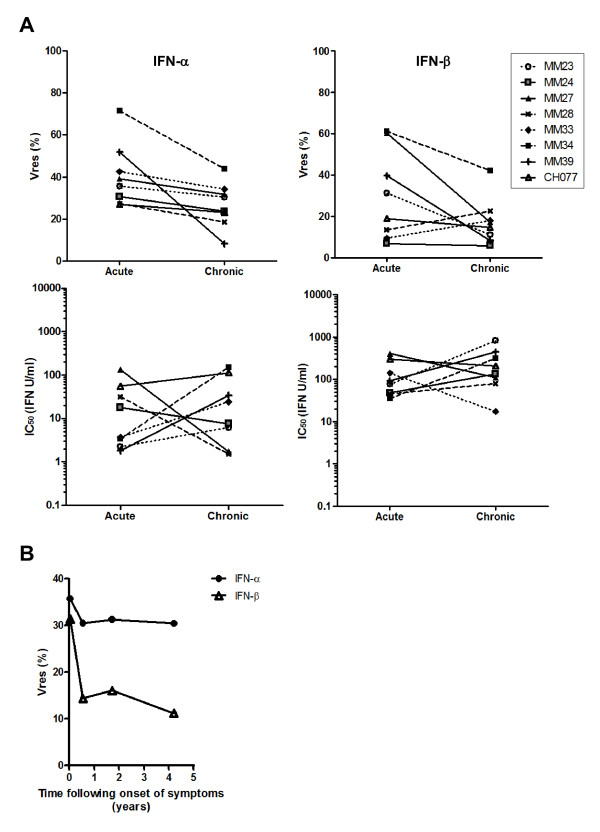
**Comparison of the type 1 IFN resistance of virus isolates generated from HIV-infected individuals during acute and chronic, asymptomatic infection. (A)** IFNα (left panels) and IFNβ (right panels) Vres (upper half of figure) and IC_50_ (lower half of figure) values of pairs of virus isolates generated from the same subjects at time-points in acute infection and chronic asymptomatic infection (after 2–7 years of chronic, untreated infection). **(B)** IFNα (filled circles) and IFNβ (open triangles) Vres values of virus isolates generated from subject MM23 in acute infection and at the indicated time-points during chronic, untreated infection. The data shown in **(A)** and **(B)** are the mean of results from a minimum of 2 independent IFNα or IFNβ assays performed with each virus isolate using different donor PBMC mixes.

To examine whether HIV-1 IFN resistance declines after the acute phase of infection as replication proceeds in the presence of low levels of on-going type 1 IFN production, the IFN-resistance of viruses derived at a time-point during chronic, asymptomatic infection from 8 non-ART-treated HIV-infected individuals (patients CH077, MM23, MM24, MM27, MM28, MM33, MM34 and MM39, Table 1) was compared to that of matched virus isolates generated from the same subjects during acute infection. As shown in Figure 4A, the IFNα Vres values of acute-infection virus isolates were significantly higher than those of the virus isolates generated from plasma cryopreserved from the same subjects after 2–7 years of chronic, untreated infection (p = 0.024, paired t-test). In patients infected with viruses that were relatively resistant to control by IFNβ, the IFNβ Vres values of the viruses isolated from plasma during chronic infection were also lower than those of the matched virus isolated during acute infection, although as the virus isolates generated from some subjects during acute infection were very sensitive to control by IFNβ, the IFNβ Vres values of the acute and chronic time-point-derived virus isolates did not differ significantly. Interestingly, although acute time-point-derived virus isolates were able to replicate significantly better in the presence of maximally-suppressive concentrations of IFNα than viruses isolated from the same patients during chronic infection, there was no significant difference in the IFNα (or IFNβ) IC_50_ values of the two groups of virus isolates (Figure 4A). These results are consistent with the hypothesis that IFNα plays an important role in control of HIV replication during the establishment of infection, and furthermore suggest that high concentrations of IFNα may be present at sites of early virus replication.

Virus isolates were generated from different subjects at chronic infection time-points ranging from approx. 2 to 7 years post-infection. Analysis of the relationship between the time-point during chronic infection at which virus isolates were generated and their IFNα or IFNβ Vres values revealed a trend for virus isolates generated earlier in chronic infection to exhibit lower levels of IFNα resistance than those generated at late time-points. Moreover, there was a significant correlation (Spearman r = 0.7857, p = 0.0279) between the number of days following the onset of symptoms (DFOSx) of the acute retroviral syndrome at which chronic virus isolates were generated and their IFNβ Vres values (not shown). This suggests that viral resistance to control by type 1 IFNs does not decline continuously throughout chronic infection, but rather that the initial decline in IFN resistance occurs within the first 2 years post-infection, and that IFN resistance may begin to increase as disease progression occurs. Consistent with this idea, prior studies have shown that virus isolates derived from chronically-infected subjects who had progressed to AIDS were more resistant to IFNα2 than viruses isolated from chronic donors without AIDS, a finding attributed to the presence of higher serum IFNα levels in the former group [81,82]. It is notable that the virus isolate generated from patient MM34 during chronic infection, which exhibited the highest IFNα and IFNβ Vres values of all the chronic time-point viruses tested, was derived from plasma cryopreserved at > 6 years post-infection, by which time the patient’s CD4 T cell count had fallen to <350 cells/mm^3^, leading to subsequent initiation of ART.

To provide further insight into the kinetics with which viral IFN resistance declines following acute infection, additional virus isolates were generated from plasma samples cryopreserved from subject MM23 at approx. 7 and 21 months post-infection, and their IFN resistance was evaluated and compared to that of the virus isolates generated from this subject during acute infection and at approx. 51 months post-infection. As shown in Figure 4B, the decline in viral type 1 IFN resistance in this subject occurred within the first few months of infection, as the IFNα and IFNβ Vres values of the virus isolates generated at approx. 7, 21 and 51 months post-infection were all comparable.

Previous studies in which the viral quasispecies present in plasma during acute HIV-1 infection has been analysed by single genome amplification (SGA) and sequencing has shown that systemic infection in subjects infected via a mucosal route is frequently initiated by just one or a very limited number of founder viruses [2,3]: in cohorts of men who have sex with men (MSM) such as the one from which our virus isolates were derived, systemic virus infection was initiated by a single founder virus in approx. 60% of subjects [83]. The data in Figure 4 showing that viruses isolated from plasma during acute infection have a higher level of IFNα resistance than matched virus isolates derived from the same subjects during early chronic infection could therefore reflect establishment of infection by a single founder virus that subsequently acquired mutation(s) that increased sensitivity to IFN-mediated control; and/or establishment of infection by several founder viruses, with the quasispecies present in plasma during acute infection being dominated by a relatively IFN-resistant virus that was subsequently out-grown by a more IFN-sensitive virus (or recombinant). As a decline in viral IFNα resistance from acute to chronic infection was observed in all 8 MSM subjects studied, it seems likely that both mechanisms may operate *in vivo*. To explore this further, additional experiments were conducted using viruses derived from subjects in whom systemic infection was known to be established by a single founder virus.

### Comparison of the type 1 IFN resistance of molecularly cloned founder viruses and 6-month consensus molecular clones

To determine whether founder viruses derived from subjects in whom systemic infection was known to be established by a single variant exhibited a similar level of type 1 IFN resistance to virus isolates generated from the plasma of MSM subjects during acute HIV infection, and explore whether these single founder viruses acquired mutations during the first few months of infection that resulted in a decline in viral IFN resistance, further experiments were conducted using viruses derived from infectious molecular clones (IMCs) of inferred founder virus sequences and 6-month consensus (6-mo) virus sequences.

**Figure 5:**
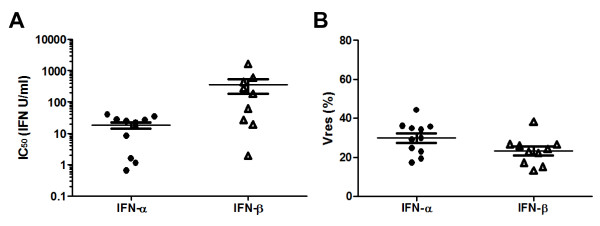
**IFN-α and IFN-β resistance of viruses generated from IMCs corresponding to the deduced founder virus sequence from subjects in whom systemic HIV infection was established by a single virus. (A)** IFNα (filled circles) and IFNβ (open triangles) IC_50_ values of founder virus IMCs from different subjects; and **(B)** IFNα (filled circles) and IFNβ (open triangles) Vres values of these founder virus IMCs. Each datapoint represents the mean (of results obtained in a minimum of 2 independent IFN inhibition assays) IC_50_ or Vres value calculated for a given virus. The horizontal lines show the group mean IC_50_ or Vres values, plus and minus one standard error.

First, to address the IFN resistance of viruses that had been subject to a stringent transmission bottleneck, we studied founder IMC viruses from 11 subjects in each of whom systemic HIV infection had previously been shown to be established by a single founder virus (patients CH040, CH058, CH077, CH162, CH164, CH185, CH236, CH264, CH470, CH850 and SUMA, Table 1). The IFNα and IFNβ IC_50_ and Vres values of these viruses are shown in Figure 5A and B, respectively. As had been observed with acute time-point virus isolates, the mean IC_50_ value for IFNα (19 U/ml) was significantly lower than that for IFNβ (366 U/ml) (p = 0.015, Mann–Whitney test) indicating that IFNα is capable of inhibiting founder virus replication at lower concentrations than IFNβ; but the mean Vres values for IFNα (30%) and IFNβ (23%) did not differ significantly, suggesting that at high concentrations IFNα and IFNβ are capable of suppressing virus replication to similar extents (although there was a trend for the founder viruses to be suppressed somewhat more efficiently by maximally-inhibitory concentrations of IFNβ than IFNα). The founder viruses comprised a mixture of clade B viruses derived from MSM subjects recruited from clinical sites in the US and clade C viruses derived from female heterosexual subjects recruited from clinical sites in Africa; however no differences were observed in the IFNα and IFNβ IC_50_ and Vres values of the two sets of viruses (not shown). Importantly, the IFNα and IFNβ IC_50_ and Vres values of the founder IMC-derived viruses (Figure 5A and B) did not differ significantly from those of the virus isolates generated from MSM subjects acutely infected with HIV (Figure 2A and B), indicating that viruses isolated from the plasma during acute HIV infection and viruses generated from IMCs of inferred founder virus genomes both exhibit similar levels of IFN resistance. This was further supported by the finding that the type 1 IFN resistance of virus isolates derived from acute infection plasma samples from 3 of the subjects from whom founder IMCs had been generated (patients CH058, CH077 and CH470, Table 1) closely matched that of the subjects’ founder IMC-derived viruses (data not shown). Combining the data from all virus isolates and IMC-derived viruses tested, the mean IC_50_ values for IFNα and IFNβ were 22 U/ml and 346 U/ml respectively; and the mean Vres values for IFNα and IFNβ were 33% and 26%.

**Figure 6:**
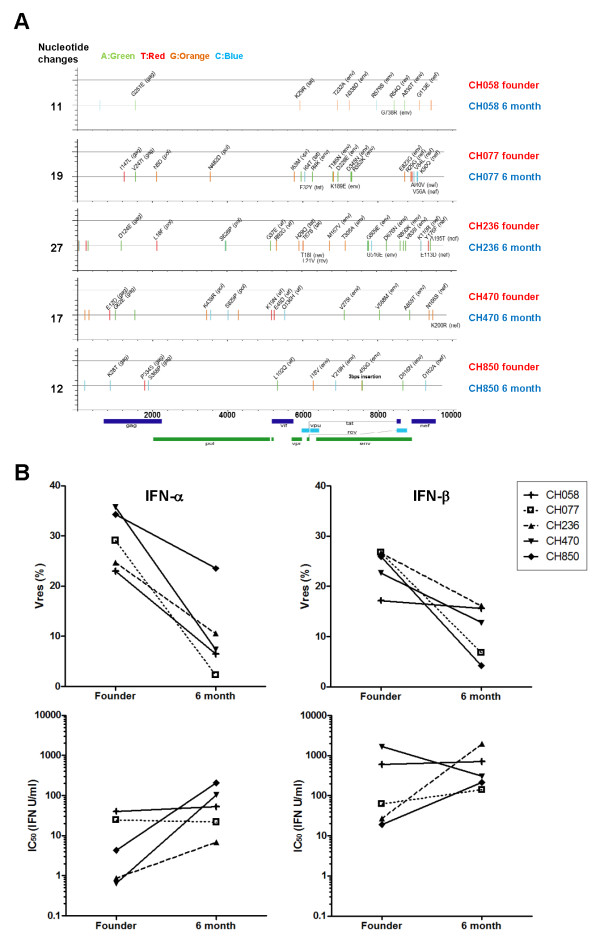
**Comparison of the type 1 IFN resistance of viruses generated from IMCs of founder and 6-month consensus virus sequences. (A)** Diagrammatic representation of the nucleotide differences between the founder and 6-month consensus (6-mo) IMC pairs from subjects CH058, CH077, CH236, CH470 and CH850. The founder and 6-mo IMC sequences from each subject are represented by horizontal grey lines. The horizontal axis indicates nucleotide positions in the alignment beginning at the start of the U3 region of the 5‘ LTR and extending to the end of the U5 region of the 3’ LTR, based on HXB2 reference sequence numbering (http://www.hiv.lanl.gov/content/sequence/HIV/REVIEWS/HXB2.html). Nucleotide differences between the founder and 6-mo IMC sequences are indicated by tic-marks on the 6-mo sequence, with the colour of the tic-mark indicating the base present in the 6 mo IMC (A in green, T in red, G in orange and C in light blue). Non-synonymous changes are labelled. The total number of nucleotide differences between each founder and 6-mo IMC pair is indicated. **(B)** IFNα (left panels) and IFNβ (right panels) Vres (upper half of figure) and IC_50_ (lower half of figure) values of viruses generated from the founder and 6-mo IMCs depicted in **(A)**. The data shown are the mean of results from a minimum of 2 independent IFNα or IFNβ assays performed with each virus using different donor PBMC mixes.

Following establishment of systemic HIV infection by a single founder virus, the plasma viral quasispecies starts to diversify and selection for viruses bearing sequence changes that confer a fitness advantage in the conditions under which virus replication is currently occurring begins. Longitudinal analysis of the plasma viral quasispecies in subjects CH058, CH077, CH236, CH470 and CH850 by SGA and sequencing ( [67,84] and unpublished data) revealed that by 6 months post-infection there were 11, 19, 27, 17 and 12 nucleotide positions, respectively, at which 50% or more of the viral sequences amplified exhibited nucleotide substitutions compared to the corresponding founder virus (Figure 6A). Most of these were non-synonymous changes, some of which were shown to confer escape from recognition by epitope-specific T cell responses or antibody responses in the newly-infected recipient, whilst others were hypothesised to represent loss of fitness-impairing mutations selected to confer escape from adaptive immune responses in the virus donor [84-86]. Other changes may have been selected by other immune responses, may have been compensatory changes selected to reduce the fitness costs of immune escape mutations [87], may have enhanced viral replication by other mechanisms, or may have been bystander mutations carried along with selected mutations.

To examine whether one or more of these nucleotide changes resulted in a reduction in the type 1 IFN resistance of the founder virus, a 6-month consensus (6-mo) IMC was created for each subject by introducing all the nucleotide substitutions that occurred in 50% or more of the SGA-derived sequences at the 6-month time-point into the corresponding founder genome sequence [69]. The type 1 IFN resistance of the founder and 6-mo IMC-derived viruses was then compared. As shown in Figure 6B, the IFNα and IFNβ Vres values of the 6-mo IMC-derived viruses from all five subjects were all lower than those of the matched founder IMC-derived viruses. This difference was statistically significant for both IFNα (p = 0.005, paired t test) and IFNβ (p = 0.026, paired t test). As observed with virus isolates generated during acute and chronic infection, there was no significant difference in the IC_50_ values of founder and 6-mo IMC-derived viruses (Figure 6B). These findings provide further strong evidence that viruses that succeed in establishing systemic HIV infection are more resistant to control by type 1 IFN than viruses representative of the quasispecies present at later time-points in infection, and that viral IFN resistance can decline within a 6-month period following acute HIV-1 infection.

In future experiments, it will be important to determine which of the nucleotide differences between the founder and 6-month viruses are responsible for their differential IFN resistance and which antiviral ISG(s) these changes affect control by. Inspection of the IMC sequences (Figure 6A) indicates that almost all of the sequence changes in the five pairs of viruses occurred at distinct sites (although the CH058 and CH236 6-month IMCs each had a (different) amino acid substitution at position 29 of Tat), suggesting that diverse sequence changes are inducing differential resistance to one or more common ISGs and/or that the differential IFN resistance of paired viruses reflects differences in their capacity to antagonise different ISGs. For example, the Tat sequences of the CH058, CH077 and CH236 founder IMCs each differ by 1 or 2 amino acids from their matched 6-month IMCs, which might result in the latter activating PKR and 2’ ,5’-OAS more efficiently [33,51,52]; and the Vif sequences of the CH236, CH470 and CH850 founder IMCs each differ by between 1 and 3 amino acids from their matched 6-month IMCs, which might result in differences in their resistance to APOBEC3G/F activity [25,47]. Notably, one of the mutations in the CH470 6-mo IMC is a glutamic acid (E) to aspartic acid (D) change at position 45 of Vif, and in a previous study where the effect of natural variation in Vif on its ability to antagonise APOBEC3F and APOBEC3G activity was assessed, NL4.3 Vif bearing a E45G mutation was shown to display only weak activity against APOBEC3G [88]. Although a change from glutamic acid to aspartic acid may have less dramatic effects on Vif function than a change to a glycine residue at the same position, this mutation may potentially contribute to the enhanced IFN sensitivity of the CH470 6-month virus. Likewise, amino acid changes in other viral proteins could also affect viral sensitivity to control by other known or as yet unidentified antiviral ISGs; and it is also possible that synonymous changes, or changes in the untranslated regions, may impact on the efficiency of viral control by antiviral ISGs [89]. However, further work is required to evaluate the contribution of these and/or other changes to the differential IFN resistance of founder and 6-mo viruses.

The observation that the type 1 IFN resistance of founder viruses declines within the first 6 months of infection suggests that the sequence changes responsible for the increase in viral IFN sensitivity must confer a strong selective advantage on virus replication during subacute and/or early infection. Given that expression of a number of ISGs continues to be sustained at higher-than-normal levels after the acute phase of HIV (and pathogenic SIV) infection [90-93], it is unlikely that an increase in viral IFN sensitivity would *per se* confer an increase in *in vivo* viral replicative fitness. It is more probable that the emergence of sequence changes conferring an increase in viral type 1 IFN sensitivity is driven by unrelated selective forces, e.g. pressure exerted by adaptive immune responses, escape from which may result in an increase in viral IFN sensitivity. Many mutations selected for to confer escape from epitope-specific T cell responses have been shown to incur a cost to *in vitro* viral fitness [79,87,94-97]. As viral replicative fitness is typically assessed in primary CD4+ T cells or CD4+ T cell lines, which express baseline levels of a number of antiviral ISGs in the absence of exogenous IFN stimulation [30,98], the fitness cost associated with some of these mutations may reflect increase in sensitivity to these factors, lending support to the idea that the increase in IFN-sensitivity in early HIV infection may occur as a side-effect of viral escape from potent adaptive responses. Nonetheless, the ability of virus isolates and IMC-derived viruses to replicate in the absence of exogenously-added IFN in our assays did not correlate with their IFN resistance (not shown), indicating that IFN resistance is not directly related to viral fitness.

Our results showing that the founder viruses that succeed in establishing systemic HIV infection are significantly more resistant to *in vitro* control by IFNα than viruses present in the same subjects during chronic, asymptomatic infection lend strong support to the hypothesis that IFNα exerts potent selective pressure on HIV replication during the initial stages of infection, resulting in preferential establishment of systemic infection by relatively IFN-resistant viruses. These findings confirm and extend results from a recent study where the IFNα resistance of a panel of molecularly-cloned founder HIVs was compared to that of viruses derived from IMCs generated from unmatched chronically-infected subjects [70]. This shows that our observations are generalizable to larger numbers of acutely-infected individuals, and provides confirmation that HIV-1 infection is commonly established by relative IFN-resistant viruses. As the transmission partners of the acutely-infected individuals in our cohorts were unknown, we were not able to determine whether this finding reflects selection during the initial stages of infection for IFN-resistant viral variants from within the donor viral quasispecies and/or preferential establishment of systemic infection following transmission of virus from donors harbouring IFN-resistant viruses. This question will need to be addressed in future studies using samples from acutely-infected subjects with known transmission partners, or in macaque mucosal SIV transmission models.

The importance of type 1 IFNs in control of HIV-1 replication is supported by the fact that this virus has evolved multiple strategies for limiting type 1 IFN production by the cells it infects [38,39]. However, pDCs produce high levels of type 1 IFN following exposure to HIV virions or infected cells [42,43] (and can be repeatedly stimulated with HIV, enabling persistent IFN production [99]), as a consequence of which a robust type 1 IFN response is triggered following HIV transmission. If, as our results suggest, type 1 IFN-mediated restriction of HIV-1 replication at initial sites of infection in the mucosa and potentially also draining lymph nodes limits the ability of transmitted viruses to undergo sufficient amplification to establish a systemic infection, this raises the question of why HIV-1 has not also evolved strategies to limit type 1 IFN production by pDCs. In contrast to HIV-1, hepatitis B and C viruses elicit very little type 1 IFN production by pDCs [100,101] and acute infection with these viruses does not trigger a strong systemic type 1 IFN response [18], a “stealth” strategy that enables initial viral amplification to occur in the face of more limited immune control. The explanation for HIV’s potent pDC-stimulatory activity may lie in the fact that HIV-exposed pDCs produce not only type 1 IFNs, but also other soluble factors including beta-chemokines, which attract CD4+ T cells. CD4+ T cell recruitment to mucosal sites of HIV transmission may play a critical role in providing a substrate for initial viral amplification (particularly in the absence of pre-existing local inflammation), making it essential for HIV to retain a strong pDC-stimulatory capacity.

Previous attempts to block mucosal SIV transmission in macaques by administering TLR agonists to induce type 1 IFN production at mucosal transmission sites have not been successful [59,60], likely as a consequence of the local immune activation also triggered by these agents. Our observations supporting an important role for type 1 IFNs in restricting viral replication early after transmission provide a rationale for evaluation of the ability of local and/or systemic administration of IFNα to block the establishment of SIV/HIV infection. In addition to the potential utility of IFNα as part of a microbicide strategy to block HIV transmission, the prospect of developing vaccines to elicit a sustained or rapidly-reactivated up-regulation of antiviral ISGs at sites of early virus replication should also be explored [102].

## Conclusions

In summary, the results from this study show that the replication of plasma virus isolates generated from subjects acutely infected with HIV-1 and viruses produced from IMCs of founder HIV genomes can be partially inhibited by type 1 IFNs *in vitro*. The mean IC_50_ value for IFNα2 was lower than that for IFNβ, although at maximally-inhibitory concentrations both IFN subtypes inhibited virus replication to similar extents. Individual virus isolates exhibited differential susceptibility to inhibition by IFNα2 and IFNβ, likely reflecting differences in viral resistance to the antiviral activity of differentially up-regulated ISGs. Plasma virus isolates from subjects acutely infected with HIV-1 were significantly more resistant to *in vitro* control by IFNα than plasma virus isolates generated from the same subjects during chronic, asymptomatic infection. Following establishment of HIV infection by relatively IFN-resistant viruses, viral IFN resistance declined rapidly (within the first 6 months of infection), suggesting that this decline may occur as a side-effect of escape from adaptive responses exerting potent pressure on viral replication at this time. The establishment of systemic HIV infection by relatively IFNα-resistant founder viruses lends strong support to the hypothesis that IFNα plays an important role in control of HIV-1 replication in the initial stages of infection, prior to systemic viral spread. These findings suggest that the antiviral activity of type 1 IFNs could be employed in prophylactic and potentially also therapeutic strategies to combat HIV-1 infection.

## Methods

### Study participants

Peripheral blood samples were drawn at serial time-points during HIV infection from US, UK and African subjects enrolled in the Centre for HIV and AIDS Vaccine Immunology (CHAVI) 001 acute HIV infection study or recruited from clinics at the Mortimer Market Centre (London, UK) or the University of Alabama, Birmingham (Alabama, USA). Ethical approval for these studies was obtained from local ethics committees and all study subjects provided written informed consent. Plasma was separated by centrifugation and cryopreserved prior to use. If subjects received ART, only samples drawn prior to the commencement of therapy were employed in this study. Set-point viral loads were calculated for subjects not receiving early ART as described [65].

### Generation and expansion of virus isolates

Virus isolates were generated from HIV-infected individuals by co-culture of plasma cryopreserved during acute or chronic infection with activated CD4+ cells derived from the peripheral blood of HIV-seronegative donors. PBMCs from three different HIV-seronegative donors (isolated from leukapheresis cones purchased from the National Blood Transfusion Service, Oxford, UK) were mixed and stimulated with 0.5 μg/ml αCD3 antibody clone UCHT1 (R&D systems), 0.1 μg/ml αCD28 antibody clone CD28.2 (eBioscience) and 20 U/ml IL-2 (Sigma-Aldrich) in R20 medium (RPMI containing 300 mg/l L-glutamine (Sigma-Aldrich) supplemented with 20% fetal calf serum (FCS) (Sigma-Aldrich), 10 U/ml penicillin (Sigma-Aldrich) and 0.1 mg/ml streptomycin (Sigma-Aldrich)) for 3 days. Stimulated PBMC were depleted of CD8+ cells using CD8 microbeads (Miltenyi Biotec) according to the manufacturer’s protocol, then CD8-depleted PBMC were spinoculated (2 hours, 1200 g) with patient plasma that had been pre-incubated for 30 min with 50 ul CD44 microbeads (Miltenyi Biotec) per 1 ml of plasma. The cells were then re-suspended in R20 + 20 U/ml IL-2 and incubated at 37°C in 5% CO_2_. After 48 hours the culture was supplemented with fresh stimulated CD8-depleted PBMC by spinoculation (2 hours, 1200 g) before further culture. Virus-containing supernatants were harvested every second day for 7–14 days and their p24 content was measured using a p24 capture ELISA (Advanced Bioscience Laboratories) following the manufacturer’s instructions. ELISA plates were read using a SpectraMAX 250 plate reader with SoftMaxPro software (Molecular Devices). The infectious virus titre present in supernatants containing high levels of p24 antigen was determined by TCID_50_ assay as described below. If necessary, a further round of viral expansion was conducted to generate sufficient quantities of virus for experimental analysis.

### Generation of IMCs

The plasma quasispecies in selected patients was analysed at sequential time-points during acute and early infection by single genome sequencing (SGS) and IMCs corresponding to the inferred founder virus sequence were generated as described [67,68]. In five subjects where systemic infection had been established by a single founder virus a 6-month consensus (6-mo) IMC was also generated. SGS-derived sequences from 154–194 days post-Fiebig I/II were used to determine this consensus sequence. At polymorphic positions, the majority nucleotide was selected. At positions where there was no single nucleotide representing >50% of sequences, the most prevalent nucleotide change was selected. IMCs with this 6-mo sequence were constructed by chemical synthesis of overlapping subgenomic fragments (Blue Heron), followed by ligation and cloning as described [69]. All IMCs were sequence confirmed.

To produce viral stocks from IMCs, plasmid DNA was transfected into 293FT cells using Lipofectamine (Sigma) and virus-containing supernatants were harvested 3 days later. Where necessary, viruses were expanded by growth in CD8-depleted PBMCs for 7–14 days as described above.

### Titration of viral stocks

The infectious titre of viral stocks was determined by TCID_50_ assay. PBMC from three HIV-seronegative donors were depleted of CD8+ cells using CD8 microbeads and the CD8-depleted cells were mixed and stimulated with 2.5 μg/ml phytohaemagglutinin (PHA) (Sigma-Aldrich) and 10 U/ml IL-2 in R10 (RPMI containing 300 mg/l L-glutamine supplemented with 10% FCS, 10 U/ml penicillin and 0.1 mg/ml streptomycin) for 3 days. Cells were infected with serial dilutions of virus by spinoculation (2 hours, 1200 g) and then washed three times before re-suspension in R10 plus 10 U/ml IL-2. After 7 days supernatants were collected and assayed for the presence of virus by p24 ELISA. The TCID_50_ was calculated using the method of Reed and Muench.

### Analysis of viral IFN sensitivity

PBMC from three HIV-seronegative donors were depleted of CD8+ cells using CD8 microbeads and the CD8-depleted cells were mixed and stimulated with 2.5 μg/ml PHA and 10 U/ml IL-2 in R10 for 3 days. After washing, cells were pre-treated with IFNα2 (Peprotech) or IFNβ (Peprotech) at concentrations from 0 – 5000 U/ml in R10 for 4 hours. Cells were then plated into flat-bottomed 96-well plates at 2 × 10^5^/well, setting up 4 wells of cells pre-treated with each IFN concentration, and were infected with HIV at a moi of 0.001 TCID_50_/cell by spinoculation (2 hours, 1200 g). Following infection, cells were washed and, unless otherwise specified, the IFN-containing medium replaced, supplemented with 10 U/ml IL-2. After 7 days supernatants were removed and their p24 content was assessed by ELISA as a measure of viral replication.

Data was analysed using Excel (Microsoft) and GraphPad Prism v 5.0 (GraphPad software). To calculate the ability of the virus to replicate in the presence of maximal concentrations of IFNα and β (Vres), p24 production in cells treated with maximally-suppressive IFN concentrations was expressed as a percentage of p24 production from cells that had not been treated with IFN. The concentration of IFNα and β required to achieve 50% of the inhibition of virus replication observed at maximal IFN concentrations (IC_50_) was also calculated (Figure 1C). Each virus was tested at least twice in assays performed using mixed cells from different donors, and the results shown are the mean Vres and IC_50_ values from the replicate assays.

### Statistical analysis

Datasets were assessed for normality, and the significance of differences between groups was determined using a Mann–Whitney test. Differences in the IFN resistance of virus isolates or IMCs generated from the same set of subjects at time-points in acute and chronic infection were analysed using a paired t-test. The relationship between pairs of variables was assessed using a Spearman rank correlation test. All statistical analyses were carried out using GraphPad Prism v5.0.

## Abbreviations

2’,5’-OAS: 2’,5’-oligadenylate synthase; APOBEC: Apolipoprotein B mRNA-editing enzyme-catalytic, polypeptide-like; ART: Antiretroviral therapy; DFOSx: Days following onset of symptoms; FCS: Fetal calf serum; HIV-1: Human immunodeficiency virus type 1; IC50: 50% inhibitory concentration; IFITM: Interferon-induced transmembrane; IFN: Interferon; Vres: Viral resistance to inhibition by maximal IFN concentrations; IMC: Infectious molecular clones; IRF: interferon-responsive factor; ISG: Interferon-stimulated gene; moi: Multiplicity of infection; MSM: Men who have sex with men; pDC: Plasmacytoid dendritic cell; PKR: Protein kinase R; RIG-1: Retinoic acid-inducible gene 1; SGA: Single genome amplification; SGS: Single genome sequencing; SIV: Simian immunodeficiency virus; TAR: Transactivation response; TCID50: 50% tissue culture infectious dose; TLR: Toll-like receptor; TREX-1: Three prime repair exonuclease 1; TRIM: Tripartite motif-containing protein.

## Competing interests

The authors declare that they have no competing interests.

## Authors’ contributions

AEF-M participated in the design of the study, performed experimental work and data analysis and contributed to preparation of the manuscript; OED participated in the design of the study and performed experimental work and data analysis; TE and KP performed experimental work and data analysis; MMA-C provided one of the virus isolates studied; PP, IW and MSC recruited the study subjects and provided clinical and virological data; HD, FG, GMS, BHH, CO and JCK provided viral sequence data and/or HIV IMCs and participated in helpful discussions; and PB conceived of the study, participated in its design and drafted the manuscript. All authors read and approved the final manuscript.
